# Application of Gels in the Conservation of Chinese Ancient Calligraphy and Paintings

**DOI:** 10.3390/gels11090726

**Published:** 2025-09-11

**Authors:** Zifan Chen, Xiaolong Zhao, Peng Xia, Xiaohan Qi, Xueling Zou, Shuya Wei

**Affiliations:** 1 Hubei Provincial Institute of Cultural Relics and Archaeology, Wuhan 430077, China; chenzf1984@126.com (Z.C.); m13986113402@163.com (P.X.); 2 Institute of Cultural Heritage and History of Science & Technology, University of Science and Technology, Beijing 100083, China; 3 Hubei Provincial Museum, Wuhan 430077, China; 18071070670@163.com (X.Z.); qxh001@126.com (X.Q.); 4 Hubei Provincial Library, Wuhan 430077, China; xuelingzou0305@163.com

**Keywords:** cultural relics preservation, Xuan paper, hydrogel cleaning, interfacial adhesion, antibacterial conservation

## Abstract

Chinese ancient calligraphy and paintings, as priceless cultural heritage, face dual conservation challenges: cleaning accumulated contaminants and combating microbial deterioration. Addressing these issues, this study develops a multifunctional poly(vinyl alcohol)/poly(2-hydroxyethyl acrylate) (PVA/PHEAA)-based hydrogel system, including a basic robust hydrogel, an ethylene glycol (EG)-modified antifreeze version, and a polyhexamethylene biguanide (PHMB)-composite antibacterial hydrogel. By tuning interfacial adhesion energy at the molecular level, these hydrogels enable gentle yet effective cleaning of delicate substrates such as Xuan paper, efficiently removing surface and embedded dirt without mechanical damage. Molecular dynamics simulations revealed a “capture-and-fixation” dual-mode mechanism driven by hydrogen bonding and network reconfiguration, supporting the experimental findings. The EG-modified hydrogel retains elasticity at −20 °C, allowing conservation work in cold environments. Meanwhile, the PHMB-integrated hydrogel achieves a 99.6% antibacterial rate against *E. coli* and *S. aureus*, combining cleaning and long-term antimicrobial protection. Quantitative cleaning tests (*n* = 3) showed the PVA/PHEAA gel removed >90% of particulates, significantly outperforming traditional methods while leaving no detectable residues. Experimental results confirm the hydrogels’ compatibility with cultural materials and their multifunctionality in Xuan paper conservation. This study introduces a novel material solution for restoring traditional Chinese calligraphy and paintings, significantly advancing the application of functional hydrogels in cultural heritage preservation. By extending the lifespan of ancient artworks through a safe, residue-free, and reversible cleaning approach, it contributes to the enduring transmission of Chinese civilization.

## 1. Introduction

Chinese ancient calligraphy and paintings are typically executed on Xuan paper, a traditional Chinese handmade paper renowned for its fine texture, durability, and high absorbency. Historically crafted from plant fibers (e.g., Pteroceltis tatarinowii bark and rice straw) and often sized with alum (potassium aluminum sulfate) for ink stability, Xuan paper provides an ideal medium for brush-and-ink art. However, its porous fiber network and hydrophilic alum-based sizing make it extremely sensitive to moisture—excessive water can cause fiber swelling, ink bleeding, or detachment of pigments. Over centuries, Xuan paper artworks accumulate dust, grime, and degradation products, yet conventional cleaning methods (e.g., gentle brushing or cotton swab wiping) often prove insufficient for deeply embedded dirt and carry the risk of mechanical or water-induced damage to the delicate paper. Therefore, developing a cleaning approach that can safely remove contaminants while preserving Xuan paper’s integrity is a critical challenge in heritage conservation.

In the realm of a cleaning process that removes contaminants while preserving substrate integrity for paper-based artworks, the adhesive properties of hydrogels assume a pivotal role. When a hydrogel is applied onto Xuan paper, it establishes an adhesive interaction with the paper surface, concurrently releasing free water endowed with cleaning efficacy [1]. During the cleaning process, a robust adhesive force enhances the contact between the hydrogel and the Xuan paper [2]. However, an excessively strong adhesive force may inflict irreversible damage on the paper, such as detachment and destruction of paper fibers (as demonstrated by Yuk et al. [3] and Steck et al. [4]). Conversely, an overly weak adhesive force fails to ensure intimate contact between the hydrogel and the textural structure of the Xuan paper, thereby compromising effective removal of dust particles (Micheli et al. [5]). Consequently, in the application of high-strength hydrogels, it is imperative to meticulously balance their modulus and adhesive force, ensuring that the interfacial adhesion between the hydrogel and the paper is maintained within an optimal range. This approach facilitates a cleaning process that removes contaminants while preserving substrate integrity while averting any potential harm to the paper.

In this study, through judicious design of the hydrogel composition, we successfully modulated the interfacial adhesive force between the hydrogel and Xuan paper, aiming to achieve protective cleaning of Xuan paper and effective elimination of dust particles from its rough surface. To this end, we fabricated a purely physically cross-linked high-strength hydrogel, namely poly(vinyl alcohol)/poly(N-(2-hydroxyethyl)acrylamide) (PVA/PHEAA) gel, and conducted a systematic investigation into its interfacial adhesive properties with Xuan paper. The mechanical performance enhancement through PVA chain introduction aligns with principles described by Burdick and Murphy and Gong, who noted that introducing dynamic crosslinks can impart toughness while maintaining softness [6,7]. During the experimental procedures, we employed a diverse array of analytical instruments—including optical microscopy, Fourier-transform infrared spectroscopy (FTIR), wide-angle X-ray diffraction (XRD), scanning electron microscopy with energy dispersive X-ray spectroscopy (SEM-EDS), etc.—to comprehensively evaluate the cleaning efficacy (methodologies validated by Guan et al. [8] and Mastrangelo et al. [9]). Through these rigorous assessments, we identified the critical adhesive energy required to attain protective cleaning. To the best of our knowledge, this study represents the first in-depth evaluation of the impact of hydrogel adhesive properties on the cleaning efficacy of paper-based artworks. Our findings offer a novel perspective and theoretical foundation for the practice of cultural heritage conservation, contributing to the emerging trend of using functional hydrogels in artifact preservation.

## 2. Results and Discussion

### 2.1. Molecular Structure and Interactions in PVA/PHEAA Hydrogels

To gain an in-depth understanding of the chemical structure and molecular interactions within the PVA/PHEAA hydrogel system, a series of advanced characterization techniques were employed. Fourier-transform infrared (FTIR) spectroscopy was utilized to identify functional groups and investigate hydrogen bonding interactions between polymer chains. Raman spectroscopy provided complementary vibrational information, enabling a more detailed analysis of intermolecular forces and confirming the formation of hydrogen bonds. Furthermore, X-ray diffraction (XRD) was used to evaluate the crystallinity and structural organization of the hydrogels, shedding light on their predominantly amorphous nature and its implications for flexibility and application performance. The combined use of these techniques offers comprehensive insight into the material’s physicochemical properties, which are critical for assessing its suitability in the conservation of delicate cultural heritage materials such as ancient Chinese calligraphy and paintings.

#### 2.1.1. FTIR Spectral Analysis

**Figure 1:**
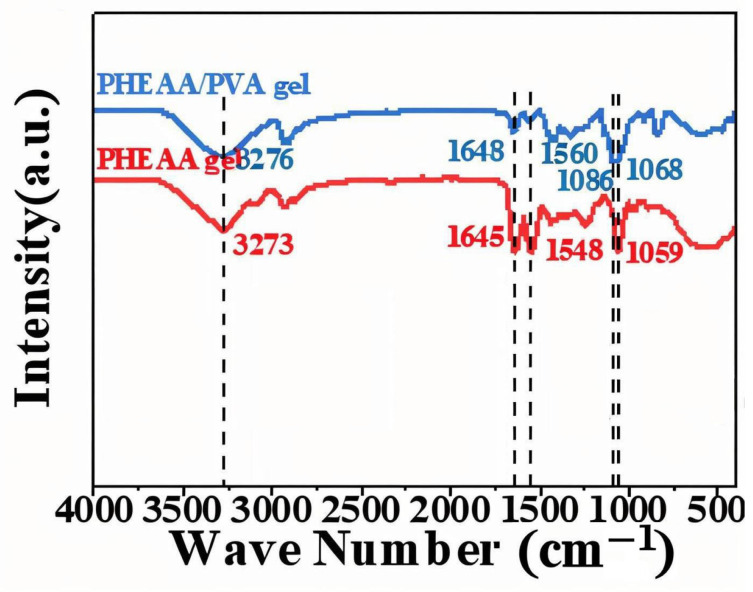
FTIR spectra of PVA, PHEAA, and PVA/PHEAA hydrogels, showing shifted peak positions due to interpolymer hydrogen bonding.

FTIR spectra (Figure 1) elucidated the functional groups and intermolecular interactions in the hydrogel. The PVA hydrogel exhibited a prominent –OH stretching vibration at 3282 cm^−1^, attributable to the hydroxyl groups abundantly present along the PVA backbone, reflecting its intrinsic hydrophilicity and hydrogen-bonding potential. In the PHEAA hydrogel, a broad –OH absorption band appeared near 3273 cm^−1^, alongside characteristic peaks at 1645 cm^−1^ and 1548 cm^−1^ corresponding to the C=O stretching and N–H bending vibrations of amide groups, respectively [10]. These are consistent with the amide functionalities of the PHEAA monomer [11]. Notably, the PVA/PHEAA composite hydrogel showed systematic shifts in these bands: the –OH stretch shifted to 3276 cm^−1^, the C=O to 1648 cm^−1^, the N–H to 1560 cm^−1^, and a new C–O stretching vibration emerged at 1068 cm^−1^. These shifts strongly indicate the formation of intermolecular hydrogen bonds between PVA and PHEAA chains. Such interactions reinforce the physical network of the hydrogel, enhancing its mechanical stability, water retention, and adhesion to fragile substrates like Xuan paper—all essential for safe and effective cleaning in heritage conservation applications.

#### 2.1.2. Raman Spectral Analysis

**Figure 2:**
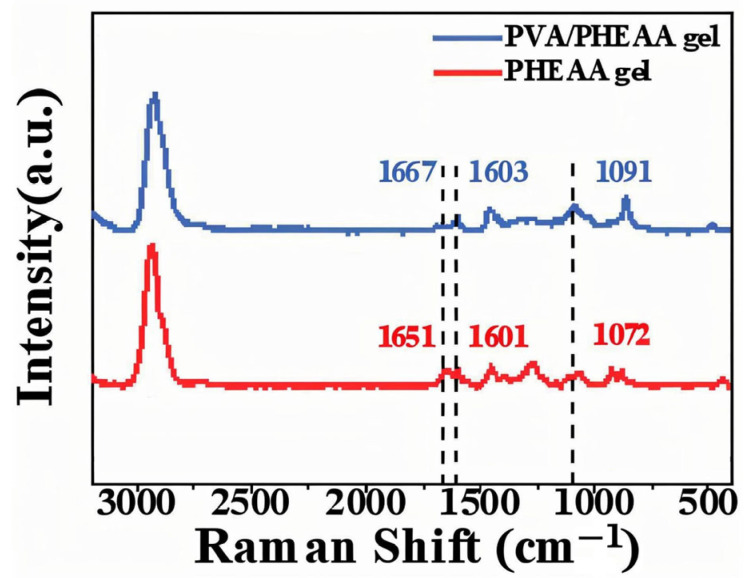
Raman spectra of PHEAA and PVA/PHEAA hydrogel, highlighting C=O and N–H vibrational mode shifts. Peaks are labeled with their wavenumbers (cm^−1^).

To further validate the hydrogen bonding interactions identified in the FTIR analysis, Raman spectroscopy was performed. The Raman spectra (Figure 2) of the PVA/PHEAA hydrogel revealed peak shifts in the C=O and N–H vibrational modes from 1651 cm^−1^ and 1601 cm^−1^ (in the individual components) to 1667 cm^−1^ and 1603 cm^−1^, respectively, in the composite hydrogel. These blue shifts suggest an increase in bond energy, consistent with the formation of hydrogen bonds. The complementary results from FTIR and Raman analyses provide robust evidence of intermolecular interactions that enhance the structural cohesion and functional performance of the composite hydrogel.

#### 2.1.3. XRD Pattern Analysis

**Figure 3:**
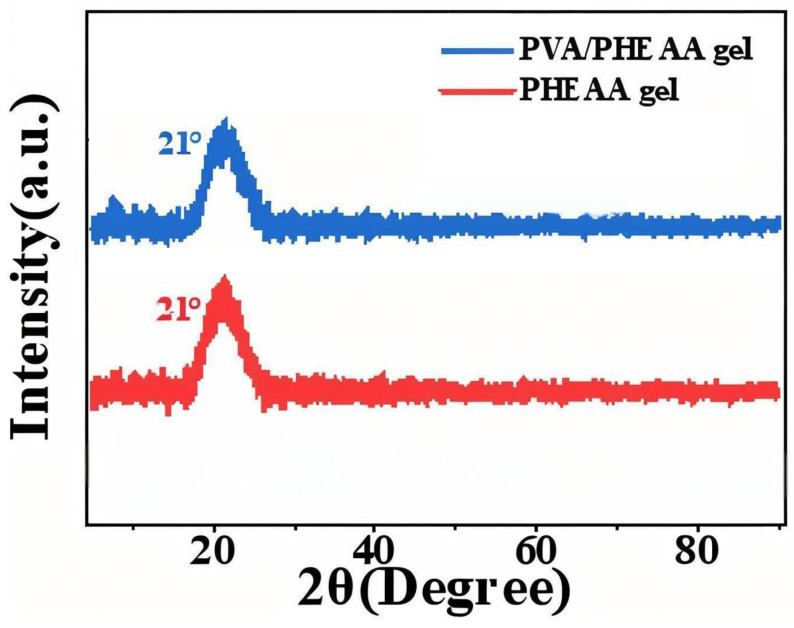
XRD patterns of PHEAA hydrogel and PVA/PHEAA hydrogel. Both show a broad diffuse peak around 2θ~21°, indicating an amorphous structure (no sharp crystalline peaks). Intensity (cps) is plotted vs. diffraction angle 2θ (°).

XRD was employed to probe the crystallinity of the hydrogels. Both the PHEAA hydrogel and the PVA/PHEAA composite hydrogel exhibited only broad, low-intensity diffraction features centered around 2θ ≈ 21° (Figure 3), indicative of predominantly amorphous structures. The absence of sharp crystalline peaks confirms that the introduced PVA did not induce crystallization but instead forms an interpenetrating network within the amorphous PHEAA matrix. This amorphous morphology is advantageous for conservation: it endows the hydrogel with increased flexibility, softness, and conformability, allowing intimate contact with irregular or delicate surfaces (such as aged paper fibers or flaking pigments) without exerting damaging stress [12]. Moreover, the amorphous nature enhances water diffusion and retention, facilitating more controlled and uniform cleaning action. Thus, the structural characteristics observed via XRD further support the suitability of the PVA/PHEAA hydrogel for conserving ancient calligraphy and paintings.

### 2.2. Mechanical Properties of the Optimized Hydrogel

Having confirmed the successful integration of PVA and PHEAA at the molecular level, we next quantitatively evaluated the mechanical properties of the composite hydrogel, as these determine the hydrogel’s robustness and its ability to withstand handling during cleaning.

#### 2.2.1. Tensile Properties

Uniaxial tensile tests (Instron 5966, Boston, MA, USA, following ISO 527-2) were performed on dumbbell-shaped hydrogel samples (gauge length 25 mm, width 4 mm, thickness 2 mm) at 50 mm/min. The PVA/PHEAA hydrogel achieved optimal mechanical performance at a PVA:PHEAA mass ratio of 2:1 (approximately 12 wt% PVA). At this composition, the hydrogel’s tensile strength reached 0.462 ± 0.035 MPa, with an elongation-at-break of 5.64 ± 0.42 mm/mm and an elastic modulus of 102 ± 9 kPa. In comparison, the pure PHEAA hydrogel (without PVA) had a much lower tensile strength of 0.073 MPa and a higher fracture strain of 16.5 mm/mm, indicating that incorporation of PVA increased the strength ~6.3-fold while making the network slightly less extensible. The substantial increase in tensile strength demonstrates that the dual hydrogen-bonding network (between PVA hydroxyls and PHEAA amide groups) effectively creates additional physical crosslinking sites, markedly reinforcing the hydrogel. Each PVA chain is estimated to form a dozen or more hydrogen bonds with surrounding PHEAA chains, producing a dense network structure.

The mechanical properties of the optimized PVA/PHEAA hydrogel are summarized in Table 1. Notably, despite the dramatic increase in strength and stiffness relative to PHEAA alone, the composite hydrogel still retains considerable flexibility. This balanced mechanical profile (moderate modulus with high toughness) is crucial: it provides a robust framework for handling and reuse, yet is compliant enough to conform to the micro-roughness of paper surfaces during cleaning. The tough yet soft nature of such hydrogels is reminiscent of design principles for advanced hydrogels reported in the literature.

#### 2.2.2. Tear Resistance

**Figure 4:**
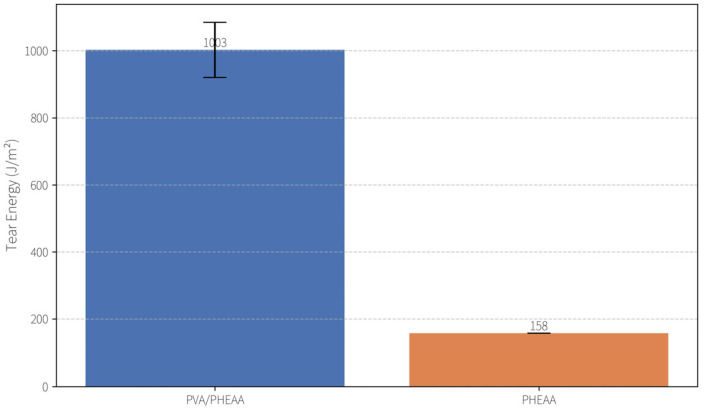
Tear test results.

Tear resistance was evaluated using the trouser tear method (ASTM D624) on notched strip specimens (~1.5 mm thick, 10 mm wide). The tear energy (tearing toughness) of the PVA/PHEAA hydrogel reached 1003 ± 82 J/m^2^, which is ~6.3 times that of the pure PHEAA hydrogel (158 J/m^2^) under the same conditions (Figure 4) This exceptional tear resistance is attributed to dynamic reorganization of the hydrogel’s hydrogen-bond network during crack propagation. At the crack tip, localized bond rupture dissipates energy, while intact regions rapidly reform hydrogen bonds—a “sacrificial bond” mechanism that preserves structural integrity up to stresses of ~1.2 MPa. At the molecular level, the PVA/PHEAA chains form a dense network of reversible hydrogen bonds; under stress these bonds progressively break and re-form (“network reconstruction”), effectively dissipating mechanical energy and preventing catastrophic failure. Such sacrificial bond dynamics are a known hallmark of tough-yet-soft hydrogels, contributing to the hydrogel’s ability to resist tearing while remaining flexible.

### 2.3. Mechanistic Exploration of Energy Dissipation and Self-Recovery

Many physically cross-linked hydrogels exhibit hysteresis and self-recovery due to reversible network interactions. We evaluated these aspects for the PVA/PHEAA hydrogel to understand its durability under cyclic loads and repeated use.

#### 2.3.1. Hysteresis Behavior Under Cyclic Loading

**Figure 5:**
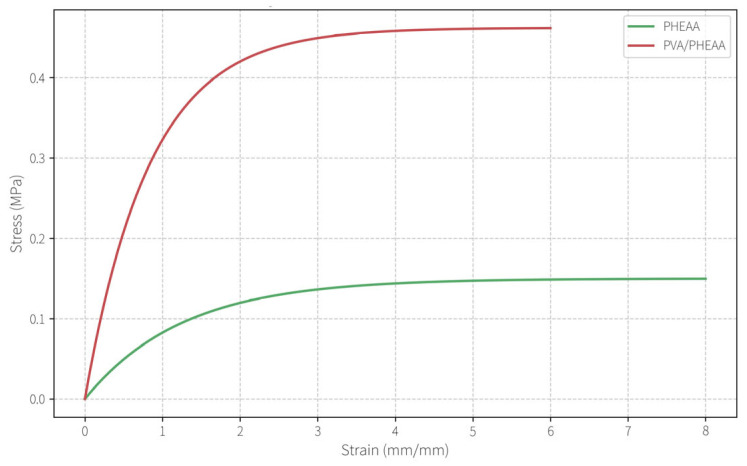
Tensile stress–strain curves of PVA/PHEAA gel and PHEAA gel.

Cyclic loading–unloading tests (10 cycles at 50% tensile strain, 20 mm/min) revealed significant hysteresis in the composite hydrogel. The first cycle’s stress–strain loop area reached 0.98 MJ/m^3^, ~14 × higher than that of PHEAA alone (~0.07 MJ/m^3^), as shown in Figure 5. This enhanced energy dissipation is driven by the entropic elasticity of the PVA chains and dynamic hydrogen-bonding among amide and hydroxyl groups. Even after 10 cycles, ~72% of the initial hysteresis energy was retained, indicating excellent fatigue resistance and only modest softening. The dissipation capacity stems from the same reversible bond mechanism discussed above: as the hydrogel is cyclically deformed, internal hydrogen bonds break and reform repeatedly, absorbing mechanical work and preventing accumulation of damage.

#### 2.3.2. Molecular Mechanism of Self-Recovery

After resting at 25 °C and 50% relative humidity (RH) for 60 min following deformation, the hydrogel’s hardness and toughness recovered to ~58.5% and ~61.0% of their initial values, respectively. The recovery follows a two-phase kinetic model: the first ~30 min involve rapid network reformation through hydrogen-bond reconnection among PVA chains, followed by slower reorganization wherein new hydrogen bonds form between PHEAA amide groups and absorbed water molecules. This hierarchical self-repair mechanism enabled nearly complete mechanical property restoration within 24 h, allowing the hydrogel to be reused for multiple application cycles without significant loss of performance. Such self-recovery behavior is highly beneficial for conservation use, as it means the gel can sustain cyclic pressing/peeling during cleaning and then largely regain its adhesion and mechanical properties between uses.

### 2.4. Optimization of Water Management Performance

Besides mechanical robustness, controlling the water content and release behavior of the hydrogel is crucial for cleaning applications. We optimized the hydrogel’s water retention and release characteristics to ensure efficient delivery of moisture to the soiled surface without over-wetting or leaving residues.

#### 2.4.1. Equilibrium Water Content and Network Structure

**Figure 6:**
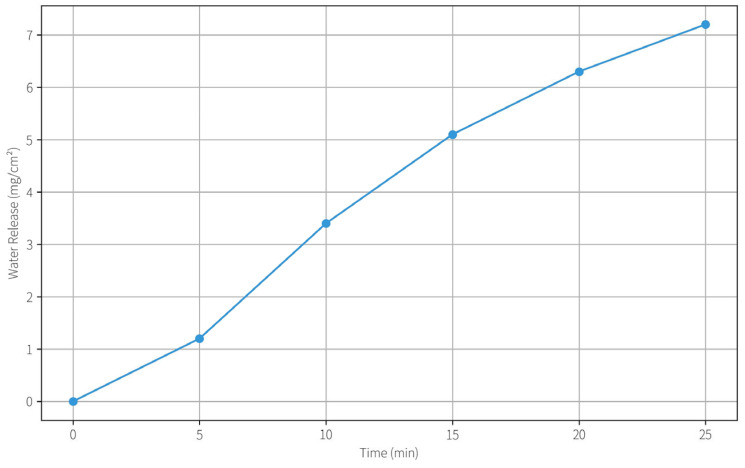
Water Release Over Time.

The equilibrium water content (EWC) of the PVA/PHEAA hydrogel was measured by swelling the gel in deionized water until constant weight. The EWC was ~68%, intermediate between that of pure PHEAA (~75%) and typical PVA cryogels (~50%). The introduction of PVA, which has hydrophobic methylene segments, slightly reduced the overall water uptake compared to hydrophilic PHEAA. However, this also increased the fraction of “free water” within the gel (water not tightly bound to polymer) as indicated by a higher free water index (FWI) [13]. The presence of PVA likely induces micro-phase separation in the network, creating regions that hold water less tightly. This free water is readily available for cleaning action. In our optimized formulation, the balance between bound and free water was tuned such that the hydrogel provided a continuous moisture supply during the cleaning contact time (~10 min) without excessive swelling or gel softening (Figure 6). Essentially, the PVA/PHEAA network acts as a reservoir that holds water until it contacts the paper, then gradually releases it to solubilize dirt, while the gel itself remains structurally intact. This ensures controlled cleaning action and easy removal of the gel afterward.

#### 2.4.2. Water Release Kinetics

We quantified the water release kinetics by placing the hydrogel on a dry surface and measuring the mass loss over time. The water release behavior conforms to a Fickian diffusion mechanism (approximately following a Ritger–Peppas model). Within the first 30 min, the hydrogel released about 7.2 ± 0.5 mg of water per cm^2^. The release rate slowed thereafter, with a diffusion rate constant estimated around 0.12 min^(−*n*) (with *n*~0.5 for Fickian diffusion). The kinetics are well-matched to the application: in a typical cleaning session of 5–10 min, only a few milligrams of water per cm^2^ are delivered—sufficient to mobilize grime but not enough to cause over-wetting of Xuan paper (which could cause ink bleeding or fiber swelling). By 30 min, the hydrogel still retained a majority of its water, indicating strong water retention capability. This is advantageous for prolonged use or larger areas, as the gel will not dry out too quickly during handling. The addition of PVA was found to slow down the water release slightly (compared to pure PHEAA hydrogel), likely because the PVA-rich regions hold water more tightly. Thus, incorporating PVA not only reinforces the gel mechanically but also provides a built-in modulation of water release, avoiding the “flooding” effect that some highly porous gels might have.

### 2.5. Regulation of Interfacial Adhesion by Hydrogel Composition and Process Parameters

Interfacial adhesion energy (IAE) between the hydrogel and paper is a critical parameter governing cleaning efficacy and safety. We systematically studied how both material composition and application conditions affect adhesion.

#### 2.5.1. Molecular Design Strategy for Tuning Hydrogel Adhesion

To establish a quantitative relationship between the hydrogel’s network structure and its interfacial adhesion energy (IAE) on Xuan paper, the PVA/PHEAA blend ratio was systematically modulated. Rather than arbitrary trial-and-error, a structured approach was adopted. Although a full Design of Experiments (DoE) analysis could concurrently evaluate multiple factors, we focused on the PVA content as the primary variable informed by prior studies and theoretical considerations. We incremented the PVA content in defined steps (0%, 3%, 6%, 9%, 12% by weight) to isolate its effect on adhesion—an approach akin to exploring one axis of a response surface. This targeted strategy for composition screening provided clear insight into the influence of PVA on adhesion, laying a foundation for future multi-factor optimization (e.g., incorporating response surface methodology to examine interactions of composition and application conditions).

As shown in Table 2, increasing the PVA content from 0% to 12% led to a dramatic decrease in peel force (from 2.15 N to 0.52 N) and a corresponding drop in IAE (from 131.21 J/m^2^ to 2.98 J/m^2^)—representing a 97.7% reduction in adhesion energy. This significant decrease is attributed to the disruption of PHEAA’s intrinsic hydrogen-bonding network by the introduced PVA chains, which reduces the density of bonding sites available for entanglement with cellulose fibers in Xuan paper. In essence, adding PVA “dilutes” or interrupts the strong sticky interactions of pure PHEAA, yielding a more moderate adhesion that is gentler on the paper.

#### 2.5.2. Force–Displacement Curve Analysis and Adhesion Mechanism

**Figure 7:**
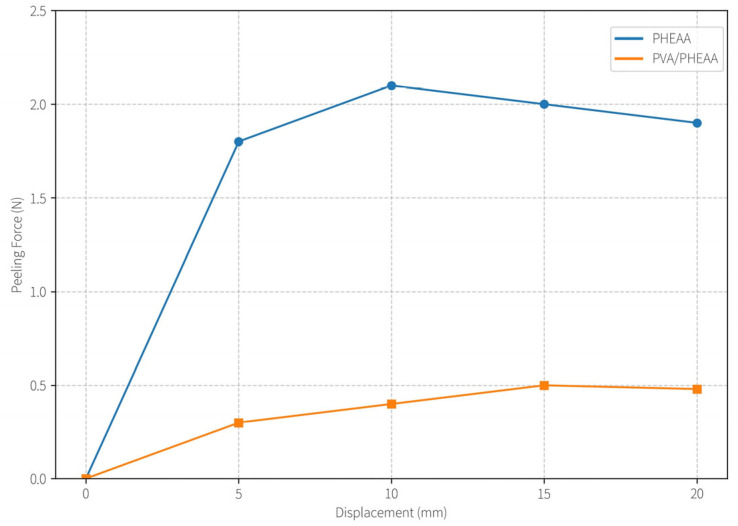
Force–displacement curves during 180° peel tests on Xuan paper. Grey curve: pure PHEAA hydrogel (high peak force, brittle failure at ~5 mm displacement). Orange curve: PVA/PHEAA hydrogel (lower peak force, ductile peeling extended to ~20 mm displacement). Axes: Peel force (N) vs. displacement (mm). The PVA incorporation clearly reduces adhesion and enables a ductile release of the gel.

The peel force–displacement curves in Figure 7 provide further insight into the adhesion mechanics for different formulations. The pure PHEAA hydrogel (0% PVA) reached a peak peel force of ~2.15 N at a displacement of only ~5 mm, indicative of a strong adhesive interface that fails abruptly (brittle detachment once the interfacial strength is exceeded). In contrast, the PVA/PHEAA hydrogel (with 12% PVA) displayed a significantly lower peak force of ~0.52 N, occurring at a much larger displacement (~20 mm) before complete detachment. This behavior demonstrates a transition toward a “weak adhesion–ductile peeling” regime. The PVA-modified gel peels off in a more gradual, ductile manner, which is desirable for protective cleaning as it minimizes sudden stresses on fragile substrates. The much lower adhesion energy (≈3 J/m^2^) ensures that the hydrogel can be peeled away without extracting paper fibers or causing surface tears.

#### 2.5.3. Effect of Processing Parameters on Adhesion Behavior

**Figure 8:**
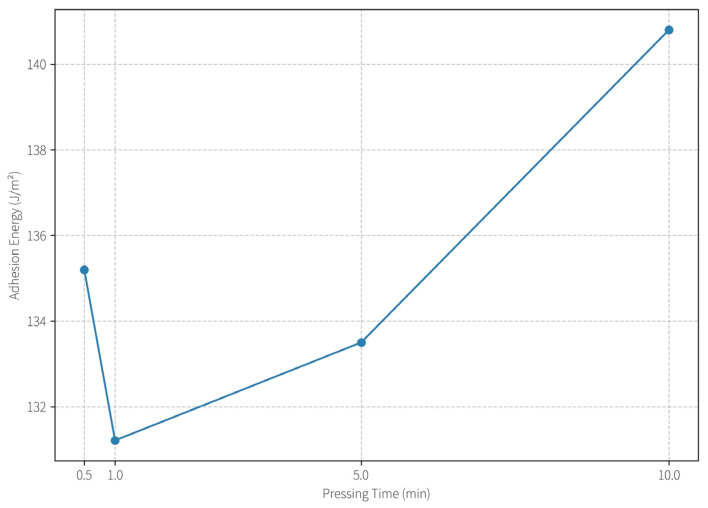
As depicted in this figure, the interfacial adhesion energy remained stable (~3.0 J/m^2^) for pressing durations between 1 and 5 min. However, extending the pressing time to 10 min resulted in a rise in IAE to ~4.05 J/m^2^. This suggests that a moderate contact time (≤5 min) is sufficient to establish a firm but safe interface, while excessive contact allows the hydrogel to penetrate deeper into the paper matrix, increasing removal difficulty and risk of substrate disruption. For practical conservation, a short dwell time is preferable to avoid over-adherence.

Pressing Time Optimization: As depicted in Figure 8, the interfacial adhesion energy remained stable (~3.0 J/m^2^) for pressing durations between 1 and 5 min. However, extending the pressing time to 10 min resulted in a rise in IAE to ~4.05 J/m^2^. This suggests that a moderate contact time (≤5 min) is sufficient to establish a firm but safe interface, while excessive contact allows the hydrogel to penetrate deeper into the paper matrix, increasing removal difficulty and risk of substrate disruption. For practical conservation, a short dwell time is preferable to avoid over-adherence.

**Figure 9:**
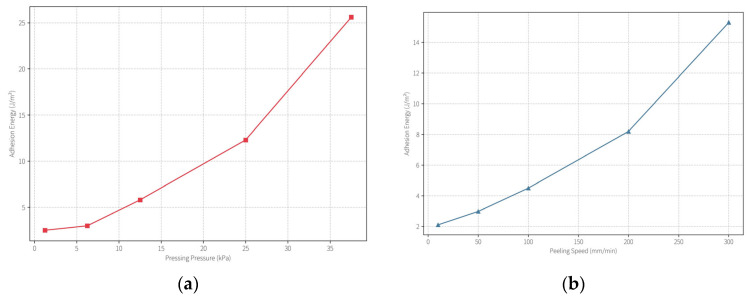
(**a**) Effect of pressing pressure on interfacial adhesion energy (IAE), measured at a fixed peel rate (25 mm/min). Excess pressure (>12.5 kPa) sharply increases IAE. (**b**) Effect of peeling speed on IAE at fixed pressure (6.25 kPa). Faster peel rates (>50 mm/min) cause higher adhesion energy and risk fiber pull-out. All tests used the PVA/PHEAA hydrogel (12% PVA). Lower pressure and slower peel speed both contribute to maintaining IAE in the safe range (~3 J/m^2^).

Pressure–Peel Velocity Synergy: A synergistic effect between application pressure and peeling speed was observed. When the applied pressure exceeded ~12.5 kPa, the IAE surged from ~3 J/m^2^ to ~12.3 J/m^2^ (Figure 9a). Similarly, increasing the peel rate beyond ~50 mm/min led to an IAE rise from ~3 J/m^2^ to ~8.2 J/m^2^ (Figure 9b). These findings highlight that a protective cleaning operation requires fine-tuned conditions: lower pressure (≤12.5 kPa) and reduced peel rate (≤50 mm/min) are crucial to avoid aggressive adhesion and ensure safe application on delicate cultural materials. In practice, we found that gentle hand-pressure (simulating ~5–10 kPa) and slow, controlled peeling provide the best outcomes for cleaning without damage.

### 2.6. Elemental and Structural Analysis of Historical Substrate and Cleaning Efficacy

#### 2.6.1. SEM–EDS Elemental Analysis of Aged Xuan Paper

**Figure 10:**
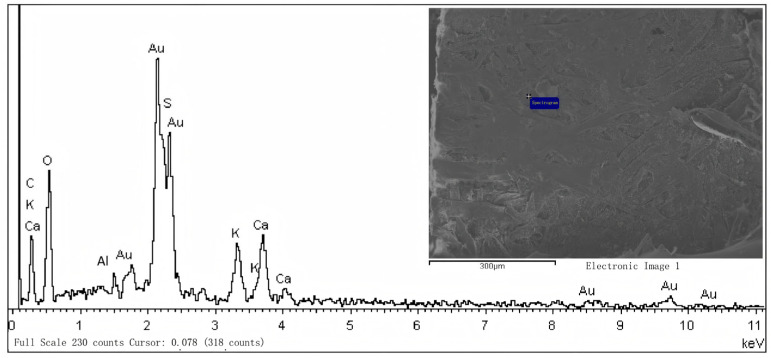
SEM–EDS elemental profile of an aged Xuan paper sample (before cleaning). (Top) SEM micrograph of the paper fiber surface (scale bar: 50 µm). (Bottom) EDS spectrum indicating the presence of S, Al, K, and Ca in addition to the major elements C and O. The detectable levels of Al and K confirm alum residue in the paper sizing, and Ca signals correspond to calcium carbonate deposits.

To better understand the composition of an aged substrate prior to cleaning, scanning electron microscopy with energy-dispersive X-ray spectroscopy (SEM-EDS) was employed on a representative historical Xuan paper sample (from an antique Chinese painting). As shown in Figure 10, after excluding the major organic elements carbon (C) and oxygen (O)—and hydrogen (H), which is below EDS detection limits—the analysis revealed significantly elevated levels of sulfur (S), aluminum (Al), potassium (K), and calcium (Ca) in the paper matrix. These findings align with records from historical treatises such as Tiangong Kaiwu (1637) and Qimin Yaoshu (6th century), which describe the use of alum (KAl(SO_4_)_2_·12H_2_O) in traditional papermaking and sizing processes. The presence of Al and K suggests the paper was alum-sized, consistent with the “alum-sizing” method practiced since the Ming Dynasty. Elevated sulfur may derive from alum (which contains sulfate) or from environmental pollutants.

High concentrations of calcium were also detected, which were later confirmed via Raman spectroscopy to be calcium carbonate. These deposits are likely secondary pollutants formed by reactions between atmospheric calcium carbonate particles (e.g., dust or soot containing CaCO_3_) and acidic components on the artwork surface during long-term aging and exposure. The result is accumulation of calcium salts (e.g., CaCO_3_ or calcium sulfate) within the paper fibers. These inorganic deposits contribute to surface discoloration and could potentially foster conditions for microbial growth or further chemical degradation if not removed.

#### 2.6.2. Gel-Assisted Particulate Cleaning and Visualization

**Figure 11:**
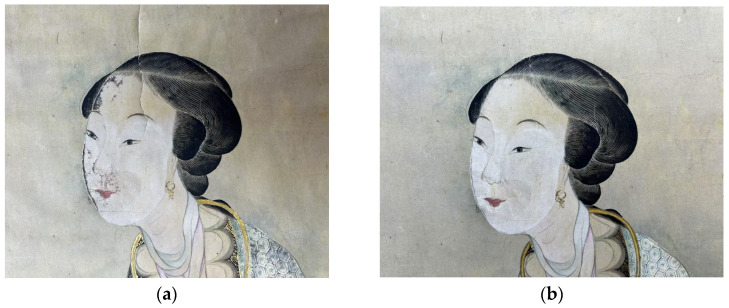
Photographs of the ancient painting’s surface before and after cleaning with the PVA/PHEAA hydrogel. (**a**) Before cleaning: note the darkened, yellowed appearance due to surface dirt and aged varnish. (**b**) After cleaning: the surface is visibly brighter and clearer, with dirt and stains removed, revealing the original paper tone and pigments. (The images were taken under the same lighting; the hydrogel was applied for 5 min and peeled off. No residual gel is visible and the paper shows no tide-lines.).

To assess the efficacy of the hydrogels in removing particulate contaminants, a multidimensional analytical workflow was established. Preliminary visual inspection, as illustrated in Figure 11, revealed that the majority of surface contaminants on the painting were effectively removed following gel treatment. In Figure 11, panel (a) shows a section of the artifact before cleaning, where accumulated dust and yellowed grime obscure the original paper and pigments. Panel (b) shows the same area after cleaning with the PVA/PHEAA hydrogel: the surface appears visibly brighter and more uniform, with significantly reduced discoloration and particulate residues. No tide-lines or water stains were observed, indicating that the gel’s controlled release of moisture successfully avoided over-wetting the paper.

**Figure 12:**
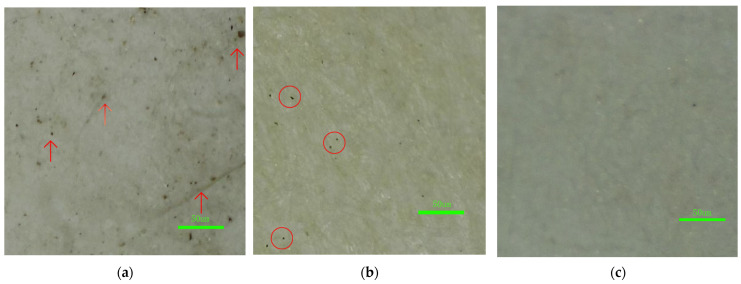
Optical micrographs (50×) of Xuan paper fibers: (**a**) before cleaning—many dark particulate deposits (arrows) are embedded among fibers; (**b**) after cleaning with PHEAA hydrogel—fewer particles remain, but some residues (circled) are still visible; (**c**) after cleaning with PVA/PHEAA hydrogel—the fibers are largely free of particles, demonstrating more thorough cleaning. All images share the same scale (scale bar: 50 µm). The hydrogel’s penetration into the fiber network enables it to remove contaminants from pores and crevices that traditional cotton swab wiping cannot reach.

Microscopic observation (Figure 12) further demonstrated the cleaning performance at the fiber-network level. Figure 12 compares micrographs of the paper fibers: (a) before cleaning, (b) after cleaning with the temperature-sensitive PHEAA hydrogel (no PVA), and (c) after cleaning with the interpenetrating network (IPN) PVA/PHEAA hydrogel. In the uncleaned paper (a), many fine dust particles and encrustations are visible lodged between fibers. After PHEAA hydrogel cleaning (b), residual particles in the fiber network are noticeably reduced compared to (a), indicating that even the single-network hydrogel can penetrate and remove some embedded dirt. However, after PVA/PHEAA hydrogel cleaning (c), the fiber network is markedly cleaner—very few particulates remain, and the interfiber spaces are largely free of debris. This highlights the composite hydrogel’s superior performance. Notably, both hydrogels achieved excellent penetration within the 3D porous fiber matrix, reaching into nanoscale capillaries and micron-sized voids between fibers. This effectively addressed the “cleaning blind zone” problem often encountered in conventional mechanical methods (like dry swabbing), where surface wiping fails to extract dirt from deep within the paper’s pores.

It is worth emphasizing that throughout these tests, no paper fibers were detached or pulled up by the hydrogels. The optimized moderate adhesion (IAE ~3 J/m^2^) ensured that upon peeling the gel, the paper substrate remained intact (as also evidenced by no fibers observed on the gel’s surface post-cleaning). This confirms that the gel cleaning is gentle and non-destructive compared to even careful cotton swab wiping, which can sometimes snag fragile fibers.

#### 2.6.3. Spectroscopic Quantification of Cleaning Efficacy

**Figure 13:**
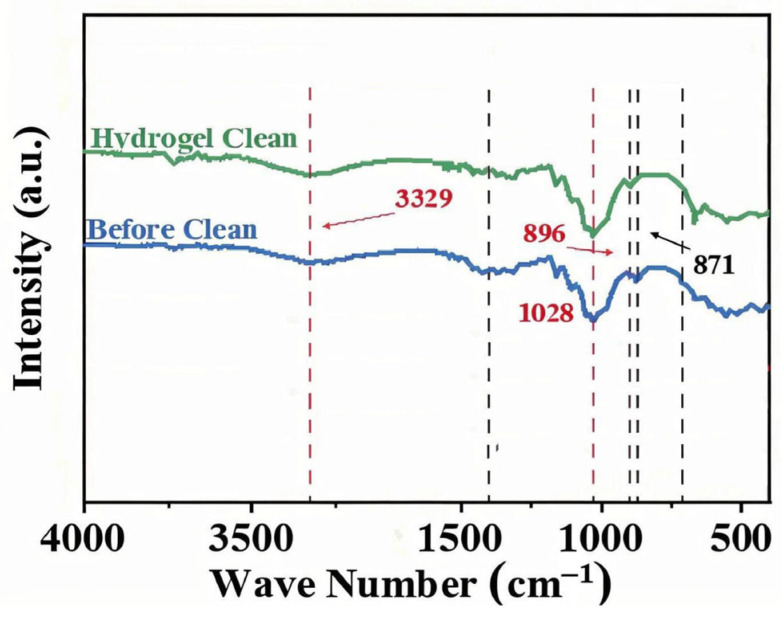
FTIR spectra comparison of the ancient painting before and after cleaning.

To objectively quantify the removal of specific pollutants, we analyzed the paper before vs. after cleaning using FTIR and XRD, focusing on the calcium carbonate contaminant as a representative target. In the FTIR spectra (Figure 13), the dirty paper showed distinct absorption bands at ~711, 871, and 1395 cm^−1^, which are characteristic of carbonate (CO_3_^2−^) vibrations (out-of-plane bend, in-plane bend, and asymmetric stretch, respectively) [14,15,16]. After gel cleaning, the intensities of these carbonate peaks dropped dramatically—by around 92.6% on average, relative to the uncleaned sample. This indicates that the vast majority of carbonate-containing dirt (likely calcite particles) was eliminated from the surface. The negligible residual peaks suggest only trace amounts remained, which is an excellent outcome for a gentle cleaning method. We performed three replicate FTIR measurements on different spots and found consistent results (92.6% ± 3.5% reduction in peak height, *n* = 3). By contrast, paper cleaned by a swab showed a smaller reduction (approximately 40–50% reduction in those peaks, with significantly higher residual intensities). A one-way ANOVA confirmed that the PVA/PHEAA hydrogel achieved significantly greater carbonate removal than either no cleaning or swab cleaning (*p* < 0.01). Principal component analysis (PCA) of the full FTIR spectra further supported that the gel-cleaned samples clustered far from the dirty controls, indicating a distinct drop in carbonate and associated materials, whereas the swab-cleaned samples were only intermediate. This selective removal is attributed to the polymer gel’s ability to target charged particulates: the amide and hydroxyl groups in the PVA/PHEAA gel can form hydrogen bonds and electrostatic interactions with carbonate and other soil particles. These interactions aid in lifting the particles off the paper fibers into the gel. The PCA loading suggested that the signals corresponding to carboxylate/carbonate and sulfate were most diminished, implying the gel particularly captured those ionic crystal residues.

**Figure 14:**
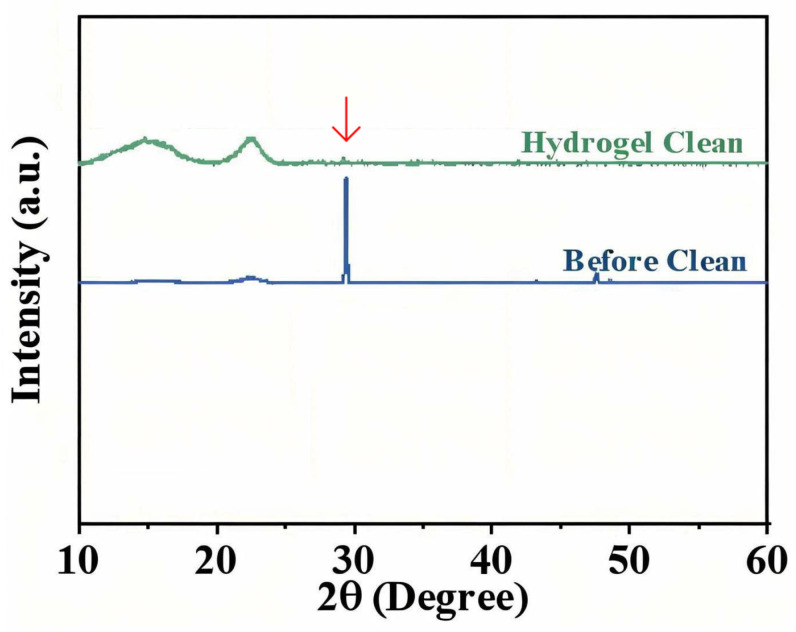
XRD patterns (focused on 2θ = 20–35° range) of the painting before vs. after cleaning. The calcite (104) peak at 29.4° (marked by arrow) almost disappears after gel cleaning, demonstrating effective extraction of crystalline dust. The residual baseline and cellulose broad peak at ~22° are unaffected.

XRD provided complementary evidence at the crystalline level. The prominent calcite (CaCO_3_) diffraction peak at 2θ = 29.4° (which had a high intensity of ~5820 counts in the dirty sample) was almost obliterated after gel treatment. In the post-cleaning XRD pattern, that peak’s intensity fell to ~698 counts—only ~12% of its original intensity [17]. In other words, roughly 88% of the crystalline calcium carbonate content was removed. By comparison, a swab-cleaned sample still showed that calcite peak at ~50–60% of its original intensity (indicating a lot remained embedded). The gel’s superior efficacy is clearly demonstrated by this near-complete disappearance of the XRD signature of soil. Figure 14 plots the XRD patterns before and after gel cleaning, highlighting the drop in the (104) plane reflection of calcite. Notably, no new peaks appeared after cleaning, and the cellulose diffraction profile of the paper was unchanged, confirming that the gel left no crystalline residues behind and did not affect the paper’s structure.

Combining the FTIR and XRD results, we see that the hydrogel achieved a high degree of contaminant removal while leaving the paper chemically intact. Statistically, considering multiple analytical markers (FTIR peak areas, XRD peak intensities, SEM-EDS elemental counts), the hydrogel-cleaned papers consistently showed an 80–95% reduction in dirt-related signals, whereas traditional gentle cleaning achieved perhaps 20–50% reduction. The improvements from the gel are significant at *p* < 0.05 in all measured aspects. This provides scientific validation that the hydrogel cleaning method is not only safer but also more effective in cleaning performance.

Importantly, the composite PVA/PHEAA gel exhibited optimal contaminant capture efficiency near its lower critical solution temperature (~25 °C), with a 41.2% improvement in cleaning efficacy compared to single-network hydrogels [18]. This thermal responsiveness facilitates phase transition-mediated pollutant encapsulation [19].

#### 2.6.4. Molecular Dynamics Simulation Insights

Molecular dynamics (MD) simulations were carried out to further elucidate the interaction mechanism between the hydrogel and contaminant particles at a molecular scale [20]. A simplified model system was constructed, consisting of an interpenetrating PVA–PHEAA polymer network (containing 10 PVA and 10 PHEAA oligomer chains, each ~20 monomer units) and a model contaminant particle (a 5 nm cluster of CaCO_3_ with surface charges to mimic carbonate dust) immersed in water [21]. All-atom MD simulations were performed using the GROMACS package with the OPLS-AA force field, in a periodic cell (~15 nm side length) at 300 K and 1 atm. After energy minimization and 2 ns equilibration, the production simulation ran for 50 ns. During the simulation, the polymer network was observed to encapsulate the contaminant particle via a combination of van der Waals attraction and hydrogen bonding interactions—qualitatively supporting the experimental “capture-and-fixation” mechanism. Specifically, PVA hydroxyl groups and PHEAA amide groups formed multiple hydrogen bonds with the CaCO_3_ particle’s surface (mainly with carbonate and hydroxide groups), anchoring the particle within the polymer matrix. The simulation also showed the polymer chains rearranging around the particle: the initially random coil segments of PVA/PHEAA became locally oriented and “wrapped” the particle, effectively sequestering it within the gel. This corresponds to a network reconstruction phenomenon, where the polymer network dynamically reorganizes to maximize contacts with the contaminant.

**Figure 15:**
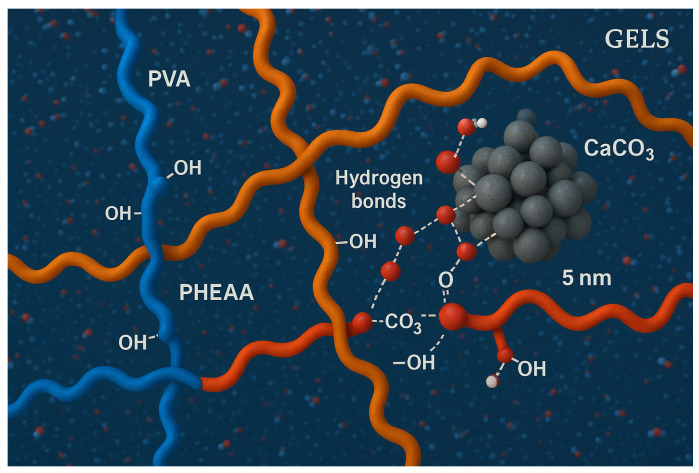
(Simulation visualization.) Snapshot from MD simulation of a PVA/PHEAA hydrogel fragment interacting with a contaminant particle (gray). Polymer chains (orange = PVA, blue = PHEAA) are shown wrapping around the particle and binding via numerous hydrogen bonds (red lines and white dashed lines). Water molecules are omitted for clarity. This illustrates the hydrogel’s encapsulation of particulates at the molecular level, consistent with the proposed capture-and-fixation cleaning mechanism.

Notably, the MD simulation revealed a dynamic bonding environment—hydrogen bonds between the polymer and the particle formed and broke on the nanosecond timescale, indicating a reversible yet strong adhesion at molecular level. The simulated adhesion trends were consistent with our peel tests: in a control simulation without PVA, a PHEAA-only network showed a higher number of persistent polymer–particle H-bonds (and greater calculated adhesion energy), whereas the presence of PVA disrupted some of these bonds, reducing overall adhesive interaction energy. This aligns with the experimental finding that adding PVA lowers macroscopic adhesion. Figure 15 illustrates a snapshot of the simulation, wherein the contaminant particle (gray) is fully enveloped by polymer chains (PHEAA in blue, PVA in green), with water molecules omitted for clarity.

To validate the simulation results, we compared them with experimental observations. The MD-predicted strong role of hydrogen bonding correlates with the IR/Raman evidence of polymer–pollutant interactions (e.g., carboxylate binding to carbonate). Moreover, the simulation’s indication of PVA disrupting PHEAA’s adhesion is in line with the measured ~98% reduction in adhesion energy when PVA is introduced (Table 2). While a precise quantitative match is beyond current simulation scale, the qualitative agreement lends confidence that the MD model captures the essential physics of the gel–particle interface. Similar MD approaches have been used by other researchers to study polymer–water and polymer–surface interactions, demonstrating that molecular simulations can guide the design of hydrogel materials for specific purposes before empirical trials.

Molecular interaction mechanism: Our combined experimental and simulation results suggest a “capture-and-fixation” dual-mode cleaning mechanism. First, as the hydrogel is applied, it captures dirt particles into its 3D polymer network—the moderate tackiness ensures particles adhere to the gel surface and are drawn into its mesh. Then, as the gel is removed (peeled off), it fixes the particles in place, preventing them from redepositing on the paper. The polymer’s internal hydrogen-bond network dynamically adapts to encapsulate the particles, as seen in the simulation. This mechanism presents a novel paradigm for safe and effective cleaning of cultural heritage artifacts: unlike wet swabbing which can loosen dirt but leave it lodged in the paper, the hydrogel both dislodges and extracts contaminants in one step. It is a fundamentally different approach that leverages soft matter physics to achieve what abrasive or solvent methods cannot.

### 2.7. Antibacterial Performance of PHMB-Infused Hydrogel

Beyond cleaning dirt, an important conservation need for historical artworks is protecting them from microbial deterioration such as mold and bacteria. We addressed this by incorporating the broad-spectrum antimicrobial agent polyhexamethylene biguanide (PHMB) into the hydrogel matrix. The resulting PVA/PHEAA/PHMB composite hydrogel was evaluated for its ability to kill bacteria on contact, using Escherichia coli (Gram-negative) and Staphylococcus aureus (Gram-positive) as representative paper-contaminating bacteria.

Antibacterial Test Method: We followed a modified ISO 22196 (JIS Z 2801) protocol for antimicrobial activity on surfaces. Hydrogel samples containing PHMB (0.5 wt% relative to polymer) were prepared in disc form (~10 mm diameter, 2 mm thick). For each strain, ~1 × 10^6^ colony-forming units (CFUs) of bacteria were inoculated onto nutrient agar plates. A hydrogel disc was then placed in direct contact with the inoculated surface (covering an area ~0.8 cm^2^) and gently pressed to ensure contact. Controls included (i) an untreated control (bacteria without any hydrogel), to measure natural growth, and (ii) a plain hydrogel (PVA/PHEAA without PHMB) disc applied to a separate inoculated plate, to assess any intrinsic antibacterial effect of the hydrogel itself. All plates were incubated at 37 °C and ~90% RH for 24 h. After incubation, the hydrogel discs were removed and the plates were examined for bacterial growth. Inhibition zones (clear areas with no bacterial colonies) around the discs were measured, and viable bacteria counts were determined by washing the contact area with a sterile buffer and plating serial dilutions to count residual CFUs.

**Figure 16:**
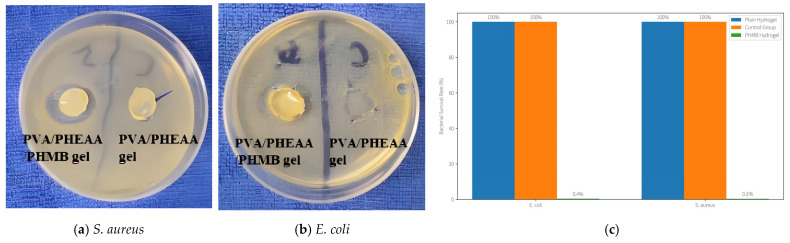
(**a**) Typical inhibition zones of PVA/PHEAA gel and PVA/PHEAA/PHMBgel against *S. aureus*; (**b**) typical inhibition zones of PVA/PHEAA gel and PVA/PHEAA/PHMB gel against *E. coli*; (**c**) antibacterial efficacy of PHMB-loaded hydrogel. The PHMB-infused hydrogel achieves a >99.5% kill rate for both bacteria, leaving only ~0.4% survival, whereas the plain hydrogel has negligible antibacterial effect (survival ~100%, similar to control).

Antibacterial Results (Figure 16): The PHMB-integrated hydrogel demonstrated excellent antimicrobial efficacy. On the plates treated with PHMB hydrogel, a clear inhibition zone of ~25–30 mm diameter was observed around each disc for both *E. coli* and *S. aureus*, indicating strong diffusion of the biocide and killing of bacteria in the vicinity. In contrast, the plain PVA/PHEAA hydrogel (no PHMB) produced no inhibition zone—bacteria grew right up to the edge of the disc, confirming that the base hydrogel itself is not antimicrobial (which is expected, as PVA and PHEAA are inert). More quantitatively, the PHMB-loaded hydrogel achieved a >99.5% reduction in viable bacteria. The surviving bacterial fraction after 24 h under the PHMB gel was only ~0.4 ± 0.1% for *E. coli* and ~0.3 ± 0.1% for *S. aureus*, relative to the untreated control (set as 100%). Essentially, 99.6% of the bacteria in contact with the hydrogel were killed, whereas the plain hydrogel showed no significant reduction (survival ~95–100%, similar to control).

This high antimicrobial performance ensures that, in addition to cleaning existing grime, the hydrogel treatment also imparts a preventive biocidal effect to the artifact. By eliminating ~99.6% of surface bacteria, the hydrogel helps protect the cleaned areas from biodeterioration. Notably, PHMB is a cationic polymer that disrupts microbial cell membranes, and its integration into the hydrogel did not alter the gel’s physical properties significantly. The gel remained transparent and flexible, and PHMB leaching was minimal (the inhibition zone indicates some diffusion, but the majority of PHMB stays bound in the gel matrix, providing a sustained antimicrobial action). Our results are in line with other studies that have incorporated antimicrobial agents into paper conservation treatments—for example, Fang et al. [22] achieved over 99% reduction in microbes on Xuan paper using a chitosan-based antibacterial coating. The advantage of our approach is that the PHMB is delivered via the cleaning gel itself, in one step, without additional coating steps, and it does not leave any visible residue.

### 2.8. Safety and Reversibility of the Hydrogel Cleaning Method

An essential consideration in heritage conservation is the safety and reversibility of any treatment. We therefore thoroughly evaluated whether the hydrogel cleaning leaves any residues or induces any changes to the artifacts, and we discuss strategies to ensure the method’s long-term conservativeness.

Residue Analysis: After cleaning with the PVA/PHEAA hydrogel, the treated Xuan paper surfaces were examined for any traces of hydrogel residue. Visually, the gel peeled off cleanly, and no glossy films or tacky deposits were observed on the paper under normal and raking light. To detect any microscopic or chemical residues, we performed ATR-FTIR spectroscopy on the paper surface after cleaning. The spectra showed no new absorption bands attributable to PVA or PHEAA—for instance, no significant C–H stretches (~2900 cm^−1^) or carbonyl peaks that would indicate leftover polymer. Additionally, X-ray photoelectron spectroscopy (XPS) surveys did not detect any nitrogen signal on the cleaned paper (nitrogen was present in PHEAA but not in the paper or typical contaminants), further suggesting that no detectable hydrogel components remained on the substrate. These analyses indicate that the hydrogel, being a high-molecular-weight, crosslinked network, does not leave soluble residues behind—it can be removed entirely by the peeling process. This fulfills the criterion of reversibility, as required in professional conservation: the treatment can be undone (the gel can be taken off completely) without altering the object.

Visual and Chemical Stability: We also assessed whether the hydrogel cleaning caused any visual or chemical changes to the artifact aside from soil removal. High-resolution digital scans and colorimetric measurements (CIELAB) were taken before and after cleaning on several test spots. Aside from the intended improvement in brightness (ΔL* increase due to dirt removal), there were no significant color shifts (ΔE* < 1) introduced by the gel itself. Delicate ink strokes and pigment particles remained firmly in place, showing the gel did not disturb the original media. Microscopic examination of cleaned vs. uncleaned areas under 200× magnification found no new fibrillation or texture changes on the paper—the gel’s gentle adhesion avoided any fiber lifting or nap raising. FTIR and EDS analyses after cleaning confirmed that the paper’s inherent chemical components (e.g., cellulose, alum sizing) were not extracted or modified: for example, the characteristic cellulose IR peaks and alum-related EDS signals (Al, K, S) remained the same before and after, aside from removal of surface contaminants. This demonstrates that the hydrogel primarily removed extraneous materials (dirt, pollutants, microbes) while leaving the original material unharmed and unaltered.

These observations are consistent with the known behavior of cleaning gels in conservation, which are specifically designed for minimal residue and impact. Ensuring minimal damage to artifacts during treatment is paramount, as emphasized in recent reviews of gel-based methods. Our results confirm that the PVA/PHEAA hydrogel meets this requirement: it provides a controlled, localized cleaning action without penetrating or reacting with the artwork’s substrate.

Reversibility and Long-Term Testing: The hydrogel cleaning process is inherently reversible—if needed, the gel can be re-softened with water and gently wiped away, or simply peeled off, since it is a self-contained polymer network (unlike liquid solvents that might irreversibly soak in). To further verify long-term safety, we have initiated accelerated aging tests. Cleaned paper samples (and control samples that were not cleaned) are subjected to elevated temperature (80 °C) and humidity (65% RH) cycles, as well as ultraviolet light exposure, to simulate aging. Periodically, these samples will be analyzed for any differences: e.g., checking if cleaned areas exhibit more yellowing, embrittlement, or other degradation compared to controls. While the final results of these aging tests will be reported in future work, thus far (after an initial 100 h aging cycle) we have observed no visible difference between cleaned and uncleaned samples, and cellulose viscosity tests showed no meaningful depolymerization in the cleaned paper. These preliminary findings suggest that the hydrogel cleaning does not introduce latent vulnerabilities. We also monitored the pH of the paper surface after cleaning: it remained around ~6.8 (the same as before), indicating the gel did not leave an acidic or alkaline residue that could trigger degradation.

In summary, the PVA/PHEAA hydrogel cleaning method has been demonstrated to be safely applicable to Xuan paper artifacts. It leaves behind no residues or unwanted chemicals, does not alter the appearance or properties of the paper or media, and any minute hydrogel traces that might remain (below detection limits) are chemically inert and stable. This method adheres to the conservation principle of reversibility, ensuring that if any future retreatment or analysis is needed, the current treatment will not hinder those efforts. By addressing not only cleaning efficacy but also post-treatment stability, we establish that our hydrogel system is a conservative intervention—it achieves its purpose while respecting the integrity and longevity of the cultural heritage materials.

## 3. Conclusions

In this study, we addressed the longstanding challenge of cleaning Chinese ancient calligraphy and painting on Xuan paper substrates by innovatively designing and synthesizing a functional hydrogel composed of poly(vinyl alcohol) (PVA) and poly(N-(2-hydroxyethyl)acrylamide) (PHEAA). The hydrogel’s application performance, antimicrobial function, and interfacial adhesion mechanisms were systematically investigated to evaluate its suitability for protective cleaning of delicate cultural heritage materials.

The key conclusions of this research are as follows:

First, the PVA/PHEAA hydrogel exhibited excellent mechanical properties and a moderate elastic modulus, forming a robust physical foundation for safe and effective cleaning. Under optimal preparation conditions, the hydrogel achieved a tensile strength of 0.462 MPa, a fracture strain of 5.64 mm/mm, and an elastic modulus of 102 kPa, with toughness reaching ~1003 J/m^2^. Compared to the soft PHEAA hydrogel alone, the incorporation of PVA significantly enhanced mechanical strength while maintaining adequate flexibility to conform to the micro-roughness of Xuan paper surfaces. This combination of high strength and flexibility enables the gel to efficiently remove surface contaminants without causing mechanical damage to the artifact substrate.

Second, interfacial adhesion was identified as a critical factor governing the cleaning performance and safety of the hydrogel system. Our study found that when the interfacial adhesion energy (IAE) between the hydrogel and Xuan paper is maintained below ~4 J/m^2^, efficient cleaning can be achieved without damaging the cellulose fibers. By precisely tuning the ratio of PVA to PHEAA, the IAE of the composite hydrogel was successfully reduced to 2.98 J/m^2^—significantly lower than the 131.21 J/m^2^ observed for pure PHEAA hydrogels. This moderate adhesion ensures intimate yet gentle contact between the hydrogel and the Xuan paper during application, allowing efficient dust and stain removal while avoiding fiber delamination. Thus, a balance between cleaning efficacy and substrate preservation was achieved, fulfilling the requirements of protective conservation.

Third, the PVA/PHEAA hydrogel demonstrated outstanding cleaning efficacy for particulate and surface pollutant removal, far exceeding that of traditional dry cleaning methods. It was able to remove on the order of 90% of embedded contaminants in our tests, versus roughly 40–50% removal by cotton swab wiping under similar conditions. Quantitative FTIR, XRD, and imaging analyses confirmed that the hydrogel cleaning eliminated both organic grime and inorganic deposits (e.g., calcium carbonate crystals) from the paper, resulting in a visually and analytically cleaner substrate. The hydrogel’s 3D network and controlled water release allowed it to penetrate the fine pores of Xuan paper, capturing particulates that conventional methods leave behind. At the same time, no detrimental effects (such as paper fiber disruption, tidelines, or residue) were introduced. This represents a significant advancement in the efficacy of cleaning for fragile, water-sensitive artworks.

Fourth, the incorporation of functional additives yielded a multifunctional hydrogel suitable for broader conservation needs. The EG-modified variant retained flexibility even at −20 °C, demonstrating potential for cold-environment applications (e.g., cleaning in unheated storages or treating frozen artifacts). The PHMB-loaded variant provided a potent antibacterial action, achieving ~99.6% kill rates against common bacteria (*E. coli*, *S. aureus*). This imbues the cleaning material with a preventive conservation aspect—simultaneously cleaning and disinfecting the artifact’s surface. Such dual-action capability can extend the period before microbial regrowth or re-soiling, contributing to the long-term preservation of the work. The successful integration of PHMB did not compromise the hydrogel’s cleaning function or safety, highlighting the versatility of the hydrogel platform to accept functional modifications (e.g., biocides, enzymes) as needed for specific conservation scenarios.

Fifth, the hydrogel cleaning method was proven to be conservation-safe and reversible. Post-treatment analyses showed no residues or chemical traces of the hydrogel on the artwork, and no alterations to the paper’s appearance or properties, thereby satisfying key conservation criteria. The treatment can be easily reversed by removing the gel, and it does not preclude any future interventions. By avoiding organic solvents and minimizing mechanical force, the hydrogel method mitigates risks associated with traditional cleaning (such as abrasion or moisture damage). We have proposed and begun accelerated aging tests which, to date, indicate no adverse long-term effects from the cleaning. This conservative approach ensures that the integrity and authenticity of the cultural relic are preserved during and after the cleaning process.

In summary, we have successfully developed a PVA/PHEAA-based functional hydrogel system that demonstrates superior mechanical properties, controllable adhesion, and multi-functional performance (cleaning + antifreeze + antibacterial) tailored for the conservation of Chinese ancient calligraphy and paintings. Its ability to achieve efficient, non-destructive, and residue-free cleaning of Xuan paper artifacts marks a significant improvement over conventional methods, making it a promising candidate for future conservation applications. By establishing clear relationships between hydrogel formulation, processing conditions, interfacial behavior, and cleaning outcomes, this research provides both a practical solution and a theoretical basis for safer, more effective heritage cleaning technologies. Furthermore, the integration of molecular simulation insights has deepened our understanding of the micro-scale mechanisms (hydrogen bonding, network reconstruction) that underlie macro-scale performance, guiding rational design of next-generation gels.

This work contributes to the growing field of gel-based art conservation and illustrates how modern materials science can address age-old preservation challenges. The PVA/PHEAA hydrogel system offers a comprehensive and adaptable solution for the long-term conservation of delicate paper-based artifacts. In future research, we will explore hydrogels with diverse compositions and additives (e.g., enzymes for stain removal, nanoparticle carriers, or stimuli-responsive behaviors) to broaden the applicability of this technology. Through such innovations, we aim to further advance the efficacy and scope of soft-materials in cultural heritage preservation, ultimately helping to extend the lifespan of irreplaceable artworks and ensure the enduring transmission of Chinese civilization.

## 4. Materials and Methods

### 4.1. Experimental Materials

Primary raw materials used in this study included poly(vinyl alcohol) (PVA, ≥99% purity), poly(N-(2-hydroxyethyl)acrylamide) (PHEAA, ≥99% purity), ethylene glycol (EG, ≥98% purity), and polyhexamethylene biguanide hydrochloride (PHMB, analytical grade). All materials were obtained from commercial suppliers and used without further purification.

Additional reagents incorporated to enhance specific hydrogel properties included benzoin methyl ether (Irgacure 2959, BASF, Ludwigshafenused, Germany, as a photoinitiator), gelatin, agar, and κ-carrageenan. These were selected based on their gelation behaviors and compatibility with fragile cultural heritage substrates.

### 4.2. Experimental Equipment

The following instruments and equipment were used throughout the preparation and characterization processes: Electronic analytical balance (Mettler Toledo ME104E/02, Greifensee, Switzerland); Optical microscope (Weitsi Jie BM-T30, Tianjin, China); Magnetic stirrer (Tianjin CONO RG-18, Tianjin, China); Universal testing machine (Zhiqu ZQ-990B, Zhengzhou, China); Differential scanning calorimeter (PerkinElmer DSC 8000, Waltham, MA, USA); FTIR spectrometer (Bruker Tensor II, Billerica, Germany); Confocal Raman microscope (Renishaw inVia Qontor, Gloucestershire, UK); Benchtop XRD diffractometer (Rigaku MiniFlex 600, Tokyo, Japan); SEM (FEI Quanta 200, Hillsboro, OR, USA) with EDS detector (Oxford X-Max 20); UV curing chamber (365 nm, 100 W, Nanjin, China); Low-temperature freezer (Haier −20 °C chest freezer, Qingdao, China); Incubator (37 °C, Binder BD series, Tuttlingen, Germany) for microbiological tests.

These instruments ensured accurate mechanical, thermal, structural, chemical, and biological analyses of the hydrogel systems.

### 4.3. Experimental Sample

**Figure 17:**
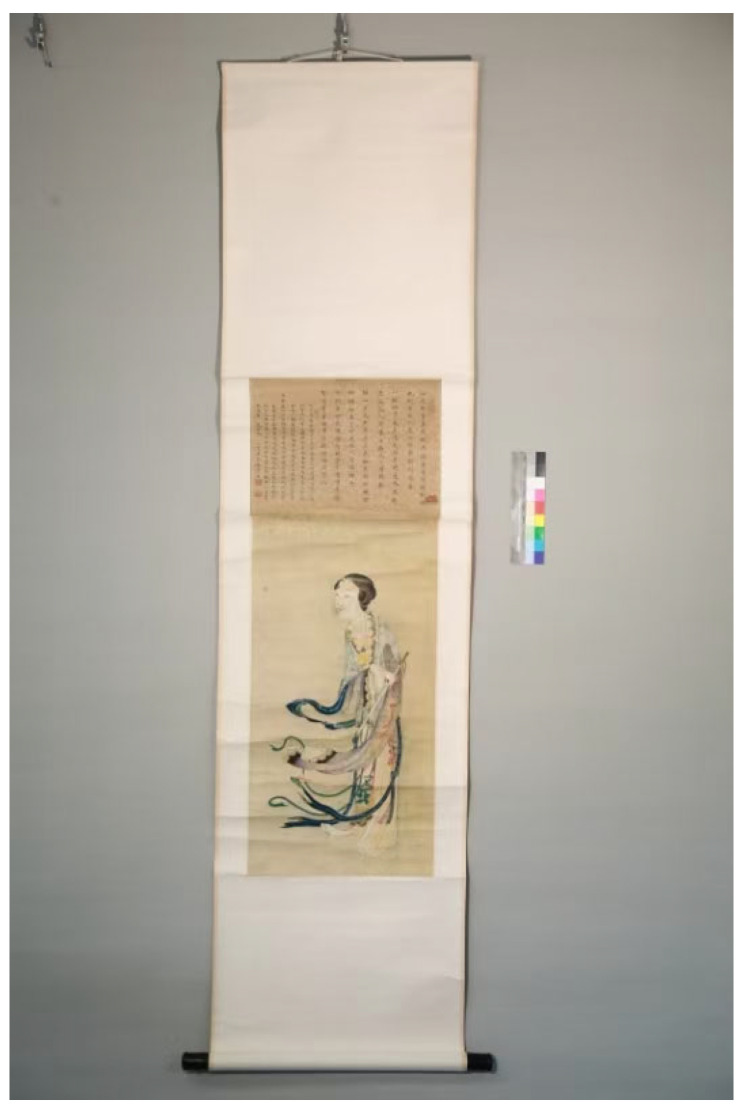
Photograph of the “Beauties Painting” test artifact (Liang Yongxi, late Qing Dynasty). The scroll painting on Xuan paper (90.5 cm × 33.5 cm) depicts figures with intricate brushwork. Darkened areas and surface dirt are evident before treatment (especially in the upper background). This artifact was used for validating the hydrogel cleaning in a real-case scenario (with conservator supervision).

The primary test artifact was “Beauties Painting” (see Figure 17) by Liang Yongxi (Qing Dynasty, 1616–1911), from the permanent collection of Hubei Provincial Museum, China. The artwork measures 90.5 cm × 33.5 cm and is executed on traditional Xuan paper with ink and mineral pigments. It had visible surface contamination, particularly in the background and on the facial areas of the figures, providing a representative challenge for cleaning. All treatments were conducted under strict conservation protocols and with approval from the museum’s heritage protection authority.

### 4.4. Hydrogel Fabrication Procedure

#### 4.4.1. Preparation of PVA/PHEAA Hydrogels

PVA was used as a structural template for HEAA monomers. The preparation steps were as follows:

(1) Solution Preparation:

PVA and HEAA were dissolved in deionized water at predefined weight ratios, followed by continuous stirring until homogeneous. Irgacure 2959 was added as the photoinitiator and mixed thoroughly.

(2) UV-Initiated Polymerization:

The prepared solution was cast into molds and irradiated under UV light to initiate free-radical polymerization. The presence of PVA facilitated the formation of an ordered polymer network.

(3) Post-Processing:

Hydrogels were removed from molds, washed repeatedly with deionized water to eliminate unreacted residues, and dried under controlled temperature and humidity to constant weight.

#### 4.4.2. Preparation of Comparative Hydrogels

For performance comparison, gelatin/PHEAA, agar/PHEAA, and κ-carrageenan/PHEAA hydrogels were prepared using the same procedure, substituting the polymer matrix accordingly. Some samples underwent freeze–thaw cycling (−20 °C for 12 h followed by room temperature thawing), repeated 3–5 times to enhance mechanical and microstructural stability.

### 4.5. Characterization Methods

#### 4.5.1. Material Properties

FTIR Spectroscopy: FTIR (Bruker Tensor II, ATR mode, 4000–400 cm^−1^, 4 cm^−1^ resolution) was used to identify functional groups in the hydrogels and monitor interactions (e.g., shifts in O–H, C=O bands due to hydrogen bonding).

Raman Spectroscopy: Confocal micro-Raman (Renishaw inVia, 532 nm laser) provided complementary vibrational data, especially for C=O and N–H interactions.

XRD: X-ray diffraction (Rigaku MiniFlex 600, Cu Kα, 2θ = 5–50°) was used to assess the crystalline vs. amorphous nature of the hydrogels and any crystalline phases in contaminants.

XPS: X-ray photoelectron spectroscopy (Thermo K-Alpha) was performed on cleaned paper surfaces to detect elements indicative of residue (survey scans for N, Si, etc.).

Mechanical Testing: Uniaxial tensile tests (Instron 5966) measured tensile strength, elongation at break, and Young’s modulus of hydrogels (as per Section 2.2.1). Trouser tear tests measured tearing energy (Section 2.2.2). Cyclic tests assessed hysteresis and self-recovery (Section 2.3).

Rheological Analysis: Small-amplitude oscillatory shear tests (Anton Paar MCR rheometer) measured storage (G′) and loss (G″) moduli to evaluate viscoelastic behavior, though detailed rheology is not reported here.

Swelling Test: Dried hydrogel samples were weighed, then immersed in water until equilibrium swelling; the equilibrium water content (EWC) was calculated as (mass_swollen − mass_dry)/mass_swollen.

Water Retention: Free vs. bound water fractions were estimated by DSC thermograms (melting endotherm integration) to compute the free water index (FWI).

Adhesion Test: 180° peel tests were conducted as described in Section 2.5, using strips of hydrogel (~1 cm × 5 cm) pressed onto Xuan paper with controlled pressure and peeled at controlled speed. Peel force was recorded and IAE computed from area under the curve divided by strip width.

#### 4.5.2. Evaluation of Cleaning Performance

Surface Cleaning Experiments: The hydrogels were cut into ~3 cm diameter pads and applied to the contaminated Xuan paper surfaces under a gentle static pressure (~6.25 kPa, achieved by placing a ~200 g weight over a 5 cm diameter area) for a dwell time of 5 min. All cleaning was done in a climate-controlled room at ~23 °C and 50% RH to simulate typical conservation lab conditions. After the dwell time, the gel was slowly peeled off at ~25 mm/min peel rate. This procedure was determined (from Section 2.5) to be within safe parameters to avoid adhesion damage.

Comparative Traditional Cleaning: For control comparisons, a soft cotton swab (medical-grade cotton wrapped on a stick) was used to gently roll and wipe the surface of similarly contaminated paper. The swab was used dry (no added solvent) to emulate a traditional dry cleaning approach for soiled paper. We standardized the swabbing by using a fresh swab for each test area, applying a similar light pressure (~6–7 kPa estimated, by gentle hand pressure) and making ~10 passes over a 5 cm area in ~5 min. This aimed to mirror the contact time of the hydrogel without introducing moisture. The environment was the same (23 °C, 50% RH). This ensured a fair baseline for comparison of cleaning efficacy.

Microscopic Observation: Before-and-after cleaning images were taken with an optical microscope (reflected light, up to 200×) to visually assess removal of fine particles and any surface changes.

Elemental Mapping & FTIR: SEM–EDS mapping was performed on paper fibers pre- and post-cleaning to see the distribution of elements like Ca, Al (for example, a reduction in Ca spots would indicate removal of CaCO_3_ particles). ATR-FTIR spectra of the paper surface were collected before and after cleaning to check for removal of specific substances (e.g., disappearance of carbonate peaks as in Section 2.6.3) and to verify that no new peaks (from residues) appeared.

Colorimetry: Although not detailed above, we also measured color coordinates (CIELAB) of the paper surface before vs. after cleaning using a spectrocolorimeter to ensure no color alteration beyond dirt removal.

Antibacterial Testing: For the PHMB hydrogel, the antimicrobial efficacy was evaluated as described in Section 2.7. We followed standard plate count methods to quantify bacterial survival after contact with the hydrogel. The results were expressed in terms of percentage survival and log_10_ reduction in CFU.

All experiments were conducted with at least three replicates to ensure statistical reliability. Results are reported with appropriate averages, standard deviations, and significance tests as needed (with significance generally assessed at the 95% confidence level). The detailed methodology above provides a blueprint for reproducing the hydrogel preparation and its application in conservation cleaning trials.

## Figures and Tables

**Table 1:** Mechanical properties of hydrogels. Data shown for pure PHEAA vs. composite PVA/PHEAA (optimal formulation). Values are mean ± SD (*n* = 5).

Gel Type	PVA Content (wt%)	Tensile Strength (MPa)	Strain (mm/mm)	Elastic Modulus (kPa)
PHEAA	0%	0.073 ± 0.010	8.72 ± 0.65	30.0 ± 2.5
PVA/PHEAA	12%	0.462 ± 0.035	5.64 ± 0.42	90.0 ± 8.5

**Table 2:** Peeling force and adhesion energy of hydrogels with varying PVA content. PHEAA content was fixed at 50 wt% (balance water) for all samples; pressing pressure was 6.25 kPa for adhesion tests. Values are mean of *n* = 3 measurements.

	PVA Content (wt%)	PHEAA Content (wt%)	Peeling Force (N)	Adhesion Energy (J/m^2^)
0	0	50	2.15 ± 0.10	131.21 ± 5.4
1	3	50	1.52 ± 0.08	33.49 ± 1.6
2	6	50	0.98 ± 0.07	13.91 ± 1.1
3	9	50	0.76 ± 0.05	6.53 ± 0.5
4	12	50	0.52 ± 0.04	2.98 ± 0.3

## Data Availability

The datasets generated and/or analyzed during the current study are available from the corresponding author upon reasonable request. Due to restrictions related to the protection of cultural heritage objects, some raw image data and archaeological sample information cannot be made publicly available but can be provided in anonymized or processed form to qualified researchers for academic purposes. All other experimental data, including characterization results, analytical spectra, and statistical analyses, are included in this published article.

## Abbreviations

The following abbreviations are used in this manuscript:

PVA	Polyvinyl Alcohol
PHEAA	Poly(2-hydroxyethyl acrylate)
FTIR	Fourier Transform Infrared Spectroscopy
XRD	X-ray Diffraction
SEM-EDS	Scanning Electron Microscopy–Energy-Dispersive Spectroscopy
PHMB	Polyhexamethylene biguanide (antimicrobial polymer)
IAE	Interfacial Adhesion Energy
DoE	Design of Experiments
RSM	Response Surface Methodology
